# Betulinic Acid Selectively Increases Protein Degradation and Enhances Prostate Cancer-Specific Apoptosis: Possible Role for Inhibition of Deubiquitinase Activity

**DOI:** 10.1371/journal.pone.0056234

**Published:** 2013-02-12

**Authors:** Teresita Reiner, Ricardo Parrondo, Alicia de las Pozas, Deanna Palenzuela, Carlos Perez-Stable

**Affiliations:** 1 Geriatric Research, Education, and Clinical Center and Research Service, Bruce W. Carter Veterans Affairs Medical Center, Miami, Florida, United States of America; 2 Division of Gerontology and Geriatric Medicine, Department of Medicine, University of Miami Miller School of Medicine, Miami, Florida, United States of America; 3 Sylvester Comprehensive Cancer Center, University of Miami Miller School of Medicine, Miami, Florida, United States of America; Mayo Clinic, United States of America

## Abstract

Inhibition of the ubiquitin-proteasome system (UPS) of protein degradation is a valid anti-cancer strategy and has led to the approval of bortezomib for the treatment of multiple myeloma. However, the alternative approach of enhancing the degradation of oncoproteins that are frequently overexpressed in cancers is less developed. Betulinic acid (BA) is a plant-derived small molecule that can increase apoptosis specifically in cancer but not in normal cells, making it an attractive anti-cancer agent. Our results in prostate cancer suggested that BA inhibited multiple deubiquitinases (DUBs), which resulted in the accumulation of poly-ubiquitinated proteins, decreased levels of oncoproteins, and increased apoptotic cell death. In normal fibroblasts, however, BA did not inhibit DUB activity nor increased total poly-ubiquitinated proteins, which was associated with a lack of effect on cell death. In the TRAMP transgenic mouse model of prostate cancer, treatment with BA (10 mg/kg) inhibited primary tumors, increased apoptosis, decreased angiogenesis and proliferation, and lowered androgen receptor and cyclin D1 protein. BA treatment also inhibited DUB activity and increased ubiquitinated proteins in TRAMP prostate cancer but had no effect on apoptosis or ubiquitination in normal mouse tissues. Overall, our data suggests that BA-mediated inhibition of DUBs and induction of apoptotic cell death specifically in prostate cancer but not in normal cells and tissues may provide an effective non-toxic and clinically selective agent for chemotherapy.

## Introduction

By virtue of their high proliferative capacity, cancer cells frequently respond to the accumulation of unfolded proteins or proteotoxic stress by enhancing the ubiquitin-proteasome system (UPS) in order to resist apoptotic cell death [1]. The UPS is the major cellular pathway that degrades unfolded proteins and controls the expression levels of specific proteins important in cell cycle, proliferation, and apoptosis [2]. Proteins are targeted for UPS-mediated degradation by the addition of multiple ubiquitin units (poly-Ub), which facilitates recognition and degradation by the UPS complex. Inhibition of the UPS and subsequent increase in multiple proteins is a valid anti-cancer strategy that has led to the development of bortezomib, an FDA approved drug for the treatment of multiple myeloma [3]. Clinically, however, bortezomib alone does not display substantial activity in castration-resistant prostate cancer (CRPC) and is often associated with dose limiting side effects such as neuropathy [4].

An alternative but less developed therapeutic strategy is to exploit the UPS by enhancing its activity and specificity in order to increase the degradation of proliferation and pro-survival proteins that are frequently overexpressed in cancers, i.e., oncoproteins. A feasible and clinically relevant method is to pursue the identification of small molecules that can activate UPS-mediated degradation of proteins such as androgen receptor (AR) in prostate cancer (PC). Betulinic acid (BA) is a plant-derived small molecule that can increase apoptosis in cancer cells, thus making it an attractive anti-cancer agent [5]. At present, BA is one of only two small molecules reported to directly activate chymotrypsin-like UPS activity *in vitro* [6], [7]. Another report demonstrates that BA inhibits the growth of LNCaP PC cells by selectively activating UPS-dependent degradation of AR as well as specificity protein (Sp) transcription factors that regulate VEGF expression [8]. However, the mechanisms by which BA specifically activates UPS-dependent degradation of AR and other factors are unknown.

In addition to stimulating UPS activity, another possible way BA can increase the degradation of specific proteins is by inhibiting deubiquitinases (DUBs). Reversible ubiquitination is a crucial mechanism in the regulation of the UPS and in the maintenance of many cell cycle and pro-survival proteins [9]–[11]. Recent findings indicate that DUBs play critical regulatory roles in most pathways involving Ub [9]–[11]. Approximately one hundred human DUBs fall into five classes, the best characterized being ubiquitin-specific proteases (USP) and ubiquitin C-terminal hydrolases (UCH). DUB-mediated removal of poly-Ub from key proteins renders them less susceptible to degradation by the UPS and therefore increases their levels. In fact, several DUBs are overexpressed in cancer and are considered to be oncogenes [9]–[11].

Because DUBs have a role in oncogenic transformation, recent attention has focused on the identification of small molecule inhibitors of DUBs. The idea is that inhibiting DUBs will elevate poly-Ub on oncoproteins and increase their recognition and degradation by the UPS pathway, resulting in greater apoptosis and improved drug efficacy [12]. Several small molecule inhibitors of DUB activity have been identified to increase the accumulation of poly-Ub proteins and enhance apoptosis in cancer cells, suggesting that DUB inhibitors are promising anti-cancer agents [13]–[18]. In this report, we showed that the ability of BA to increase the degradation of multiple proliferation and pro-survival proteins in PC cells was correlated to inhibition of DUBs. In contrast to PC cells, BA had no effect on DUB activity and degradation of proteins in normal cells, resulting in no toxicity. Our results suggested that the PC-specific effect provided by BA therapy was due to its ability to inhibit DUBs in cancer but not in non-cancer cells.

## Materials and Methods

### Ethics Statement

All animal studies were carried out with the approval of the Institutional Animal Care and Use Committee (protocol #6996.06 MR) of the Miami Veterans Affairs Medical Center (Association for Assessment and Accreditation of Laboratory Animal Care accredited) and conducted in accordance with the NIH Guidelines for the Care and Use of Laboratory Animals.

### Reagents

BA for cell culture experiments was purchased from A.G. Scientific; digitonin and polyvinyl-pyrrolidone (PVP) from Sigma-Aldrich; MG132, doxorubicin, and Coomassie blue from EMD Biosciences; N-ethylmaleimide (NEM) from Sigma; and trypan blue (0.4%) from Invitrogen.

### Treatment of TRAMP mice with BA

TRAMP transgenic mice (Jackson Laboratories) were identified by tail biopsy and PCR using Wizard SV Genomic DNA purification (Promega) and DNA primers PB-1 forward 5′-CCGGTCGACCGGAAGCTTCCACAAGTGCATTTA-3′ and Tag reverse 5′-CTCCTTT CAAGACCTAGAAGGTCCA-3′. BA was obtained from Ze-Qi Xu at Advanced Life Sciences and prepared as previously described [19]. Mice with palpable prostate tumors were randomly divided into experimental and control groups and injected i.p. 11 times over 14 days with BA (5 [BA5] or 10 [BA10] mg/kg body weight; n = 10 each dose) or vehicle control (n = 12). On day 15, primary prostate tumors were removed and their weights determined. An outer portion of primary prostate tumor was fixed in formalin for immunohistochemistry. TRAMP males without palpable prostate tumors were similarly treated with BA10 or vehicle control (n = 3, each group), prostates, spleen, thymus removed, and analyzed by immunohistochemistry.

### Immunohistochemistry

Immunostaining for apoptotic (cleaved caspase-3, Cell Signaling Technology; ApopTag Peroxidase In Situ Apoptosis Detection, Millipore) and proliferating (Ki67, NCL-Ki67p, Leica Microsystems; PCNA [PC10], Santa Cruz Biotechnology) cells was performed using rabbit polyclonal, mouse monoclonal, and biotinylated goat anti-rabbit/mouse secondary antibodies (Vector Laboratories), as previously described [20]. Blood vessel density was determined by immunostaining for CD-31 using a goat polyclonal antibody (M20; Santa Cruz Biotechnology) and a biotinylated rabbit anti-goat secondary antibody or for CD-34 using a rat polyclonal (RAM34, BD Biosciences) and goat anti-rat secondary antibody. The number of cleaved caspase-3 or Ki67 positive cells and CD-31 positive vessels were determined for BA10 and vehicle controls (n = 5 each group), as previously described [20]. Similarly, AR (N-20), cyclin D1 (DCS-6), and ubiquitin (P4D1) from Santa Cruz Biotechnology were immunostained; the number of AR positive cells was determined for BA10 and vehicle control prostate tumors.

### Cell lines

Human PC cell lines LNCaP, DU145, and PC3 [21] were obtained from the American Type Culture Collection (ATCC) and used within 6 months of resuscitation of original cultures. All PC cells were maintained in RPMI 1640 medium (Invitrogen) with 5% fetal bovine serum (Hyclone), 100 U/ml penicillin, 100 µg/ml streptomycin, and 0.25 µg/ml amphotericin (Invitrogen). PrEC normal human prostate epithelial cells were obtained from Lonza and maintained in PrEGM media. RWPE-1 normal prostate epithelial cells (obtained from Dr. Bal Lokeshwar, University of Miami and originally from ATCC) were maintained in Keratinocyte-SFM media (Invitrogen). Human foreskin BJ fibroblast cells (passage 24) and fetal lung fibroblasts (IMR-90, MRC-5) were obtained from Dr. Priyamvada Rai (University of Miami) and originally obtained from ATCC (CRL-2522, CCL-186, and CCL-171). BJ, IMR-90, and MRC-5 cells were maintained in DMEM medium (Invitrogen) with 10% fetal bovine serum, 100 U/ml penicillin, and 100 µg/ml streptomycin.

### BA cell proliferation assay

The CellTiter Aqueous cell proliferation colorimetric method from Promega was used to determine cell viability of PC cells in media containing BA (2.5, 5, 7.5, and 10 µM) or control (0.5% DMSO). Cell viability was normalized against the vehicle control and the data expressed as a percentage of control from three independent experiments done in triplicate.

### Drug treatments

PC cells were cultured in media containing BA (10 µM), MG132 (1 µM), docetaxel (1 nM), or DMSO control for varying times (24–72 h). BJ, IMR-90, MRC-5, PrEC, and RWPE-1 cells were cultured in media containing BA, doxorubicin (1 µM), or DMSO control for varying times (24–72 h). In all the experiments, floating and trypsinized attached cells were pooled for further analysis.

### Western blot analysis

Preparation of total protein lysates and western blot analysis was done as previously described [22]. The following antibodies were used: cleaved PARP (9541), CoxIV (4844), AKT (9272), and survivin (71G4B7) from Cell Signaling Technology; cytochrome c (7H8.2C12), Smac (612245), Bcl-xL (610211) and Rb (544144) from BD Biosciences; AIF (E-1), actin (C-11), cyclin A (H432), cyclin B1 (GNS1), cyclin D1 (DCS-6), Cdk1 (17), Cdk2 (D-12), Cdk4 (M2), p21 (C-19), p27 (C-19), E2F1 (KH95), AR (N-20), Mcl-1 (S-19), Bcl-2 (N-19), HA (Y-11), Ub (P4D1), Rb (C-15), and horseradish peroxidase-conjugated secondary antibody from Santa Cruz Biotechnology. Our preference was to use Coomassie blue staining of total protein as loading controls because drug treatments often affect the levels of typical housekeeping proteins such as actin or tubulin.

### Trypan blue exclusion assay

Treated and control PC, BJ, IRM-90, MRC-5, PrEC, or RWPE-1 cells were harvested, resuspended in PBS, diluted 1∶1 in 0.4% trypan blue, dead blue and live non-blue cells immediately counted using a hemacytometer, and the % dead blue cells determined from at least three independent experiments done in duplicate.

### Annexin-FITC/propidium iodide (PI) flow cytometry

Treated and control PC cells were resuspended in binding buffer followed by the addition of annexin V-FITC and PI (Annexin V Kit sc-4252 AK, Santa Cruz Biotechnology). After 20 min., cells were analyzed by flow cytometry using a Coulter XL flow cytometer and the percentage of annexin+ cells determined using WinMDI version 2.8 from two independent experiments done in triplicate.

### Mitochondrial protein release assay

Treated and control PC cells were resuspended in a buffer containing 100–200 µM digitonin, 20 mM Hepes, pH 7.5, 10 mM KCl, 1.5 mM MgCl, 1 mM EGTA, 1 mM EDTA, 1 mM DTT, 250 mM sucrose, and protease inhibitors (Roche) at 50 µl/1×10^6^ cells. After 5 min. on ice, cells were centrifuged 5 min and the supernatant used for western blot analysis. Digitonin is a detergent that preferentially permeabilizes plasma membrane compared to mitochondrial membrane [23].

### Flow cytometric cell cycle analysis

Propidium/hypotonic citrate method was used to study cell cycle distribution of BA treated PC cells [24]. Six to 8 samples were analyzed from at least three independent experiments and DNA distribution histograms generated as previously described [22], [25].

### Transient transfection of AR, cyclin D1 wild type and T286A mutant

CMV/AR expression plasmid was transfected into PC3 cells for 24 h using FuGene-HD (Roche) followed by BA (24, 48, 72 h) or control (24 h) treatment and AR protein analyzed by western blot. Cyclin D1 expression plasmids pBABE/cyclin D1 wild type (9050) and pcDNA/cyclin D1 T286A mutant (11182; cannot be degraded by UPS; [26]) were obtained from AddGene. These plasmids were transfected into LNCaP cells for 24 h followed by BA or control treatment for 24 h. Protein levels of transfected cyclin D1 protein was determined by western blot analysis using anti-HA and cyclin D1.

### Proteasome assay

The Proteasome-Glo Chymotrypsin-like Cell-Based Assay (Promega) was used to determine the effect of BA on proteasome activity in PC cells. Cells were treated with BA, BA+MG132, or control for 8, 24, 48, and 72 h, cell numbers determined, and proteasome activity measured with a luminometer (TD-20/20, Turner Designs). Light units were normalized to cell number (control treatment = 1) and 6–8 samples were analyzed from at least four independent experiments.

### DUB assay

The DUB-Glo Protease Assay (Promega) was used to determine the effect of BA on DUB activity in PC, BJ, IRM-90, and RWPE-1 cells. Cells were treated with BA, 1 nM docetaxel, or control for 8, 24, 48, and 72 h, lysed in DUB buffer (50 mM Tris-HCl, pH 7.5, 0.1% NP-40, 5 mM MgCl_2_, 250 mM sucrose, 1 mM DTT, 1 mM PMSF), centrifuged 10 min., and 10 µg protein used to determine DUB activity. Control lysates were pre-incubated with 4 mM NEM, a known DUB inhibitor, for 1 h before addition of substrate. Light units (control treatment = 1) from 6–8 samples were determined from at least three independent experiments. DUB activity was also measured in vehicle control (n = 4) and BA10 (n = 5) TRAMP prostate tumors.

### DUB labeling assay

Cell lysates were prepared as described above for the DUB assay, 20 µg of protein incubated with 500 ng HA-Ub vinyl sulfone (VS), an irreversible specific inhibitor of most DUBs (Boston Biochem) for 1.5 h at room temperature, and samples analyzed by western blot using anti-HA antibody to detect DUB labeling.

### Statistical analysis

Statistical differences between drug-treated and controls were determined by two-tailed Student's *t*-test (unequal variance) with *P*<0.05 considered significant.

## Results

### BA inhibits the growth of TRAMP prostate tumors by increasing apoptosis and decreasing angiogenesis and proliferation

**Figure 1:**
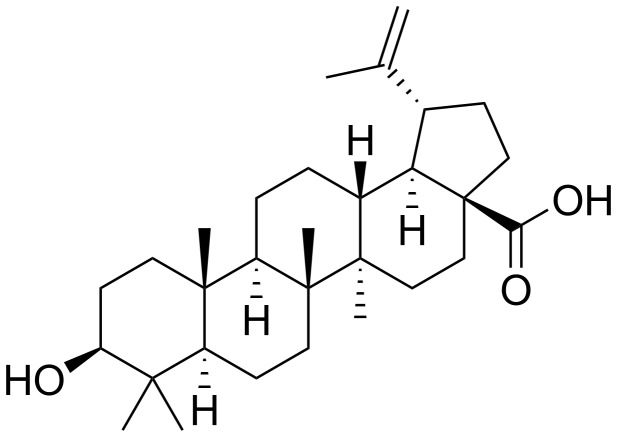
Structure of BA.

**Figure 2:**
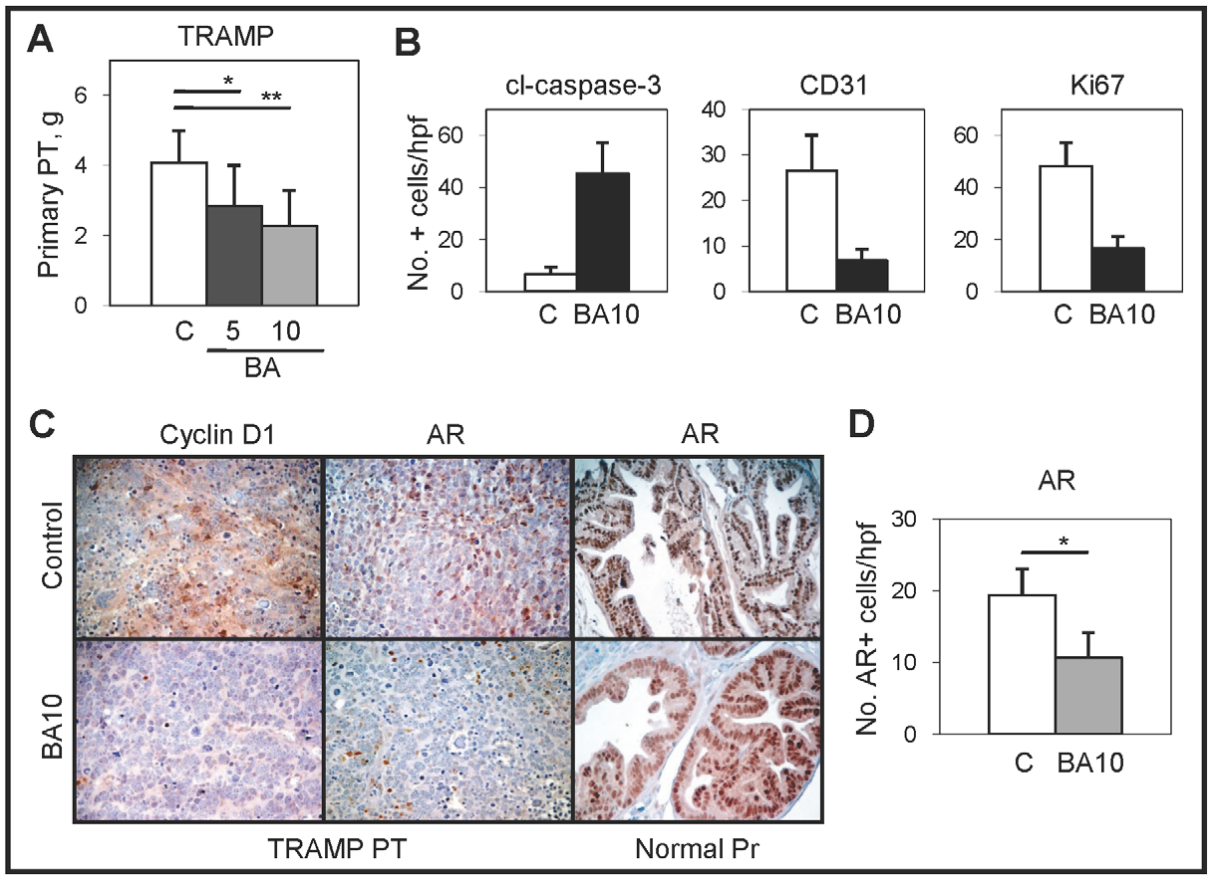
BA treatment of TRAMP mice inhibits growth of prostate tumors. (**A**) Weights of primary prostate tumors were significantly less in BA (5, 10 mg/kg) compared to vehicle control [C] treated TRAMP mice (*, *P*<0.03; **, *P*<0.002). (**B**) BA10 treatment increased cleaved caspase-3 + cells and decreased CD31 and Ki67 + cells compared to control prostate tumors. (**C**) Representative immunostaining for cyclin D1 and AR (×200) showed less protein in BA10 compared to control prostate tumors (PT). AR protein levels were similar in normal prostates (Pr) from mice treated with BA or control (×200). (**D**) Significantly decreased number of AR + cells in BA10 compared to control prostate tumors (*, *P*<4×10^−7^).

We previously utilized BA (Fig. 1) as an agent that can enhance the sensitivity of PC cell lines to cell death when combined with antimitotic agents by increasing NF-κB activity, in part due to enhanced degradation of IκBα [27], [28]. To evaluate the *in vivo* therapeutic efficacy of BA, we utilized the TRAMP transgenic mouse model of PC [29], [30]. TRAMP mice contain the prostate-specific probasin promoter linked to the SV40 T antigen oncogene, which results in the development of aggressive metastatic PC. Our results indicated that BA (5 and 10 mg/kg) significantly reduced the final weights of primary prostate tumors compared to vehicle control tumors (Fig. 2A). There were no differences in the final body weights between BA treated and control mice (data not shown). Immunohistochemistry (IHC) of cleaved (active) caspase-3, a marker for apoptotic cells, showed a significant increase in BA10 compared to vehicle control tumors (Fig. 2B and Supplementary Fig. S1A). IHC of CD31, a marker for blood vessels, and Ki67, a marker for proliferating cells, showed a significant decrease in BA10 compared to vehicle control tumors. Further confirmation using TUNEL for apoptosis, CD34 for angiogenesis, and PCNA for proliferation is shown in Supplementary Fig. S1B. These results indicated that BA induced apoptosis and inhibited angiogenesis and proliferation in TRAMP prostate tumors.

### BA decreases the levels of AR in TRAMP prostate tumors but not in normal prostate

We then sought to determine whether BA can decrease the expression levels of AR and cyclin D1 proteins in TRAMP prostate tumors. IHC and counting of AR+ cells showed a significant decrease in BA10 compared to vehicle control tumors (Figs. 2C, D). IHC of cyclin D1 also showed a significant decrease in BA10 compared to vehicle control tumors, correlating with the decrease in the Ki67 and PCNA proliferation markers (Figs. 2B, C). We next sought to determine if the BA-mediated decrease in AR also occurred in non-cancerous prostate tissue. Unlike the results in prostate tumors, the levels of AR in normal prostate were similar in BA10 compared to vehicle control, suggesting that the BA-mediated degradation of AR occurred only in tumor cells (Fig. 2C).

### BA inhibits the proliferation and increases apoptosis of PC cells

**Figure 3:**
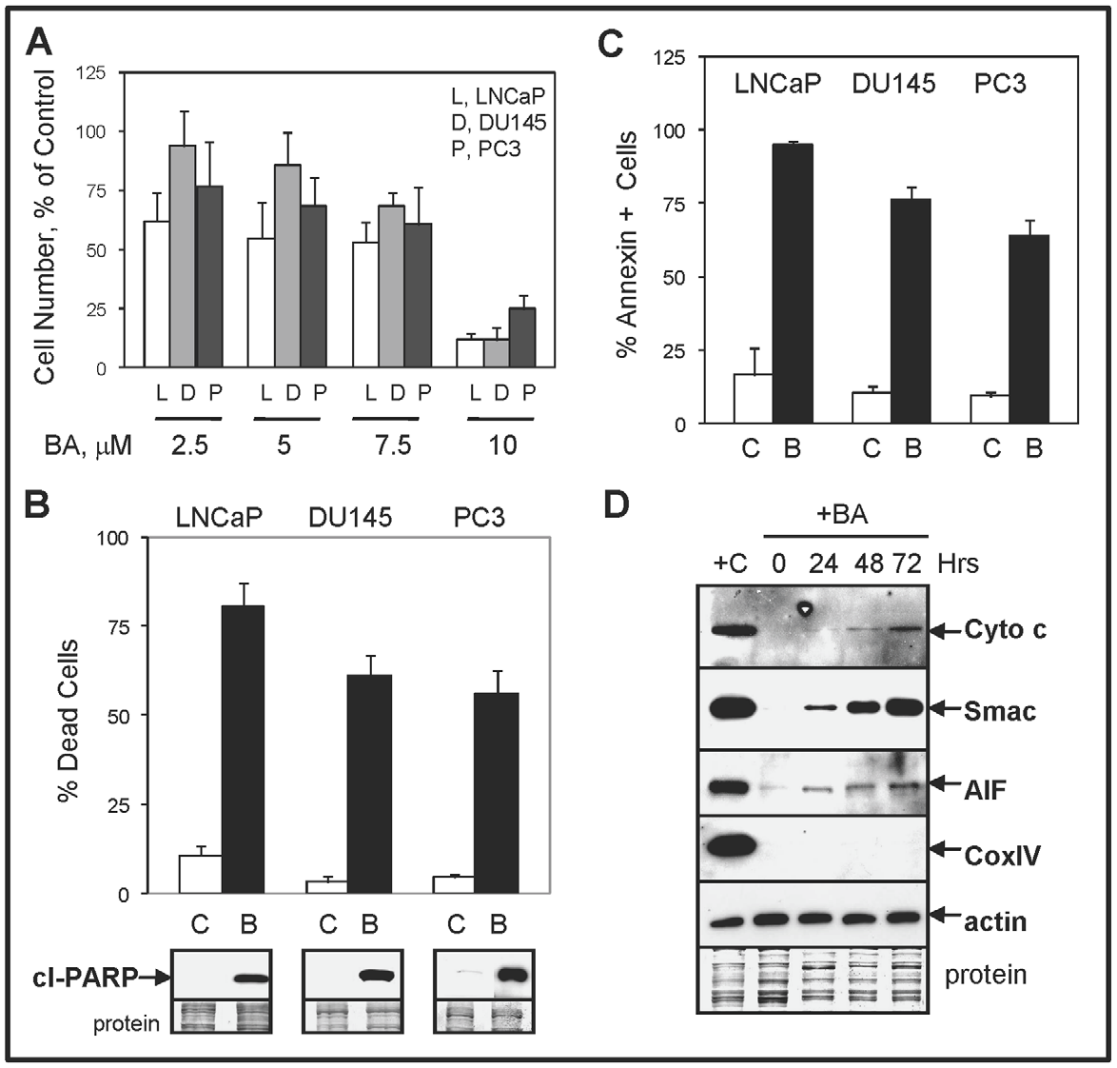
BA inhibits PC cell proliferation by inducing apoptosis. (**A**) Cell proliferation assay showed that increasing concentrations of BA (2.5 to 10 µM) inhibited LNCaP, DU145, and PC3 cells. (**B**) Trypan blue exclusion assay showed that 10 µM BA (B) increased total cell death in LNCaP (48 h), DU145 (72 h), and PC3 (72 h) compared to control (C) cells. Western blot analysis showed that BA increased cleaved (cl)-PARP levels in PC cells. Coomassie blue stain of total protein is loading control. (**C**) Flow cytometric analysis showed higher annexin-FITC stained BA compared to control treated PC cells. (**D**) Mitochondrial protein release assay and western blot showed increased levels of cytochrome c, Smac, and AIF in PC3 cells treated with BA compared to control cells. Cox IV protein was negative indicating no mitochondrial contamination and actin was the positive control. +C was lysate prepared using the standard method for total proteins.

To address the mechanisms of BA as a single agent in PC, we used androgen-dependent LNCaP and castration-resistant DU145 and PC3 cells. Using a three-day cell proliferation assay, we found that 10 µM BA inhibited the growth of all PC cells, including the more chemotherapy resistant DU145 and PC3 cells (Fig. 3A). All subsequent experiments were done using 10 µM BA. The BA anti-PC cell effect was due to increased apoptotic cell death, as determined by trypan blue exclusion assay, western blot analysis of cleaved-PARP (substrate for activated caspases), and annexin V-FITC/PI flow cytometry (Figs. 3B, C). BA is reported to target mitochondria to initiate the intrinsic pathway of apoptosis by increasing the release of mitochondrial proteins such as cytochrome c, which activates the caspase cascade [5]. Our results in PC3 cells showed that BA increased the release of cytochrome c, Smac (blocks inhibitor of apoptosis [IAP] family; [31]), and apoptosis-inducing factor (AIF; translocates to nucleus to increase DNA fragmentation; [32]) from the mitochondria (Fig. 3D). Similar results were obtained in BA treated LNCaP and DU145 cells (Supplementary Fig. S2). Thus, the BA-mediated release of pro-apoptotic proteins from the mitochondria coincided with the observed increase in apoptosis in PC cells.

### BA increases G1/S cell cycle arrest in PC cells

To investigate the cell cycle effects of BA on PC cells, we used flow cytometry analysis. Treatment of PC cells with BA for 24 h resulted in a significantly increased G1 and decreased S phase of the cell cycle, indicating a major block in G1/S (Supplementary Fig. S3A). After longer times of BA treatment, there was a significant increase in the sub-G1 cell cycle phase in all PC cells, which was reflective of greater DNA degradation that occurred in apoptotic cells (Supplementary Fig. S3B). In DU145 and PC3 but not in LNCaP there was a significant increase in G2/M after 72 h of BA treatment. These results indicated that BA induced significant disruptions in the normal cell cycle of PC cells.

### BA increases the degradation of multiple cell cycle and pro-survival proteins in PC cells

**Figure 4:**
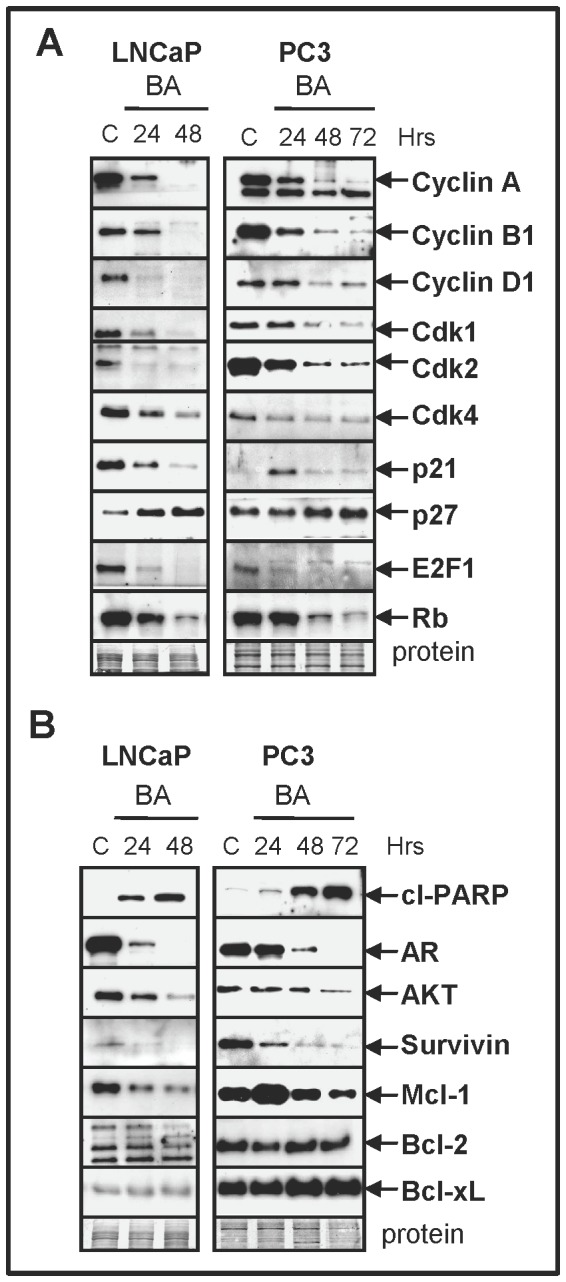
BA increases degradation of multiple cell cycle and pro-survival proteins. (**A**) Western blot analysis showed that BA treatment lowered the protein levels of cyclins, Cdks, E2F1, and Rb in LNCaP and PC3 cells. In contrast, BA treatment increased the protein levels of the Cdk inhibitor p27. (**B**) BA treatment decreased pro-survival proteins AR, AKT, survivin, and Mcl-1, which correlated with increased cl-PARP in LNCaP and PC3. AR in PC3 was from transient transfection.

Since BA is reported to increase the degradation of multiple proteins, we investigated the effect of BA on cell cycle and pro-survival proteins in PC cells by western blot analysis. Our results indicated that the protein levels of multiple cell cycle proteins including cyclins A, B1, D1; Cdks1, 2, 4; E2F1, and Rb decreased after BA treatment starting at 24 h in LNCaP and PC3 cells (Fig. 4A). In LNCaP cells, the Cdk inhibitor p21 also decreased after BA treatment but in PC3, p21 protein increased at 24 h and returned to basal levels by 48 and 72 h. In contrast, the Cdk inhibitor p27 increased after BA treatment, suggesting a role in the G1/S cell cycle block. Similar results were obtained in DU145 cells (Supplementary Fig. S4).

BA treatment increased the levels of cleaved-PARP with time in PC cells, indicating elevated levels of activated caspases and apoptosis (Fig. 4B; Supplementary Fig. S4). Interestingly, BA treatment dramatically decreased the protein levels of AR in LNCaP and in AR transfected PC3/DU145 cells. Furthermore, BA treatment decreased the levels of pro-survival proteins AKT, survivin, and Mcl-1. In contrast, BA treatment did not reduce the levels of anti-apoptotic proteins Bcl-2 and Bcl-xL (Fig. 4B; Supplementary Fig. S4). Overall, these results indicated that BA selectively increased the degradation of several proliferation and pro-survival proteins.

### BA-mediated protein degradation is dependent on the UPS

**Figure 5:**
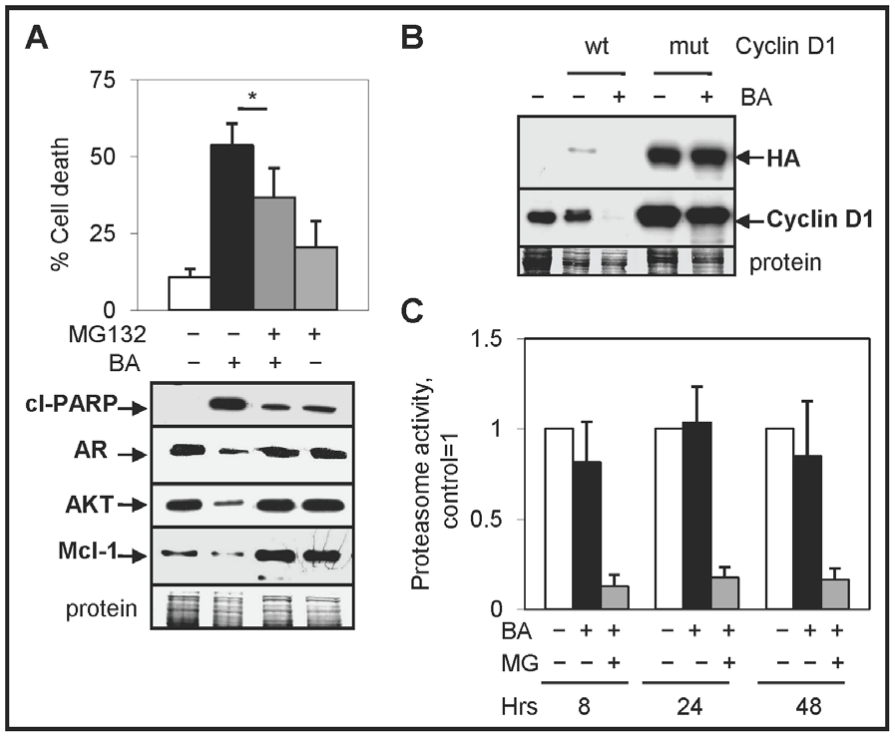
BA-mediated protein degradation is dependent on the UPS. (**A**) Trypan blue exclusion assay showed that 1 µM MG132 antagonized cell death in BA treated LNCaP cells (24 h; *, *P*<0.02). Western blot analysis showed that MG132 blocked the BA-mediated increase of cl-PARP and decrease of AR, AKT, and Mcl-1 proteins. (**B**) Western blot analysis showed that transfected cyclin D1 T286A mutant but not cyclin D1 wild type protein was resistant to BA-mediated degradation (24 h) in LNCaP cells. (**C**) Proteasome activity assay showed no significant effect in LNCaP cells treated with BA for 8, 24, and 48 h. Addition of MG132 (MG) to BA resulted in decreased activity.

Previous studies suggest that BA-mediated increase in UPS activity is a reason for enhanced protein degradation [6], [8]. Our results confirmed that in LNCaP cells, the UPS inhibitor MG132 blocked the BA-mediated decrease in AR, AKT, and Mcl-1 proteins. In addition, MG132 antagonized the BA-mediated increase in apoptotic cell death, as determined by trypan exclusion and western blot analysis of cleaved-PARP (Fig. 5A). A cyclin D1 mutant that cannot be degraded by the UPS was resistant to BA-mediated degradation, further suggesting a role for the UPS (Fig. 5B). However, our proteasome assay results showed that BA had no direct effect on UPS activity in LNCaP cells (Fig. 5C). In contrast to LNCaP cells, BA treatment of DU145 and PC3 cells resulted in significantly increased UPS activity (Supplementary Fig. S5). Overall, these results indicated that BA variably enhanced UPS activity in some (DU145 and PC3) but not all (LNCaP) PC cells.

### BA inhibits multiple DUBs leading to increased total poly-Ub proteins in PC cells

**Figure 6:**
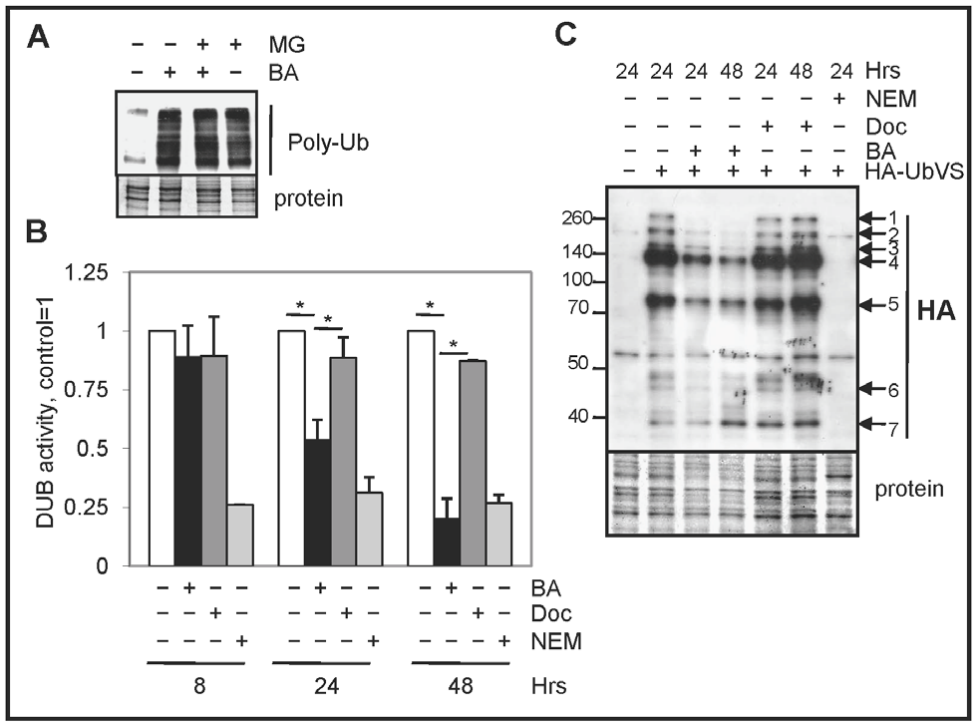
BA decreases DUB activity in PC cells. (**A**) Western blot analysis showed that BA treatment of LNCaP cells (24 h) increased total poly-Ub proteins similar to MG132 treated cells. (**B**) DUB assay showed that BA treatment of LNCaP cells significantly decreased DUB activity at 24 and 48 h relative to control treated cells ( = 1) (*, *P*<5×10^−5^). Docetaxel (Doc, 1 nM) reduced DUB activity much less than compared to BA (*, *P*<6×10^−5^). Control lysates pre-treated with 4 mM NEM for 1 h resulted in decreased DUB activity. (**C**) DUB labeling with HA-UbVS and western blot analysis with anti-HA showed that BA (10 µM) but not Doc (1 nM) inhibited multiple DUBs in LNCaP cells at 24 and 48 h. Protein bands 1–6 showed a decrease in activity with BA treatment whereas band 7 does not. Control lysates without addition of HA-UbVS or pre-incubated with NEM for 1 h were the controls. Molecular weight markers (kDa) are shown to the left. Coomassie blue stain of total protein were loading controls.

In LNCaP cells, a possible way BA can increase the selective degradation of proteins without directly activating the UPS is by inhibiting DUBs. In this case, inhibition of DUBs should result in an accumulation of total poly-Ub proteins and increase their degradation by the UPS. Our western blot results showed that BA treatment of LNCaP cells increased total poly-Ub proteins, similar to MG132 treatment (Fig. 6A). Similar results were also observed in DU145 and PC3 cells (not shown). Despite the increase in poly-Ub, it is not clear why BA treatment has no effect on UPS activity in LNCaP cells (Fig. 5C). In TRAMP prostate tumors, BA treatment also increased Ub proteins as determined by IHC and decreased DUB activity (Supplementary Fig. S6). Our DUB assay results showed that BA treatment of LNCaP cells reduced DUB activity starting at 24 h. In contrast, treatment of LNCaP with 1 nM docetaxel, a dose that increased apoptotic cell death [28], had less effect on DUB activity (Fig. 6B). Similar results were obtained with BA treatment of DU145 and PC3 cells with the exception that BA inhibited DUB activity starting at a later time (48 h) and docetaxel had a stronger DUB inhibitory effect compared to LNCaP cells (Supplementary Fig. S7). Overall, the ability of 10 µM BA to inhibit DUB activity correlated with inhibition of cell proliferation (not shown).

To further determine if BA can inhibit DUBs in LNCaP cells, we used a DUB labeling assay with HA-UbVS, a potent, irreversible, and specific inhibitor of most DUBs [33]. Since HA-UbVS only binds to active DUBs, the HA tag allows for the labeling of active DUBs present in LNCaP cells after BA treatment and analysis by western blot. Our results showed that BA treatment of LNCaP cells decreased multiple DUBs at 24 and 48 h compared to control cells (Fig. 6C). In contrast, treatment of LNCaP cells with docetaxel had no effect on DUB activity. Similar results were obtained in DU145 and PC3 cells (Supplementary Figs. S8A, B). Overall, these results suggested that BA inhibited multiple DUBs, resulting in increased poly-Ub proteins, which were likely rapidly degraded by the UPS pathway.

### BA has no effect on DUB activity in non-cancer cells

**Figure 7:**
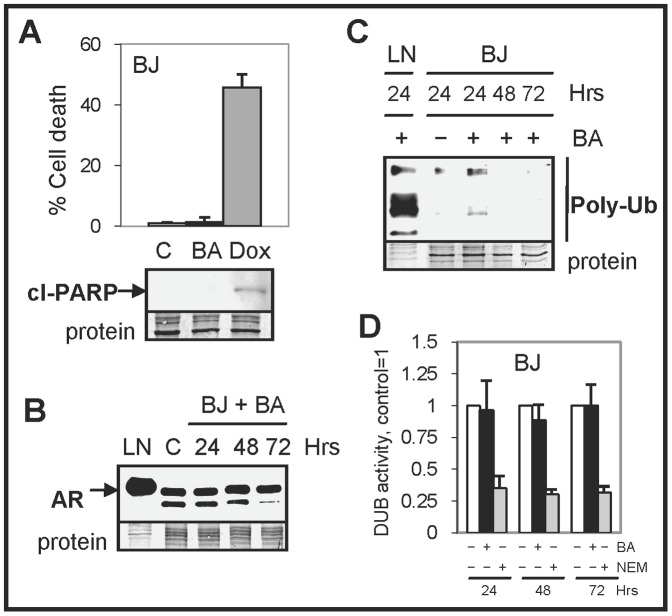
BA has no effect on DUB activity in non-cancer BJ fibroblasts. (**A**) Trypan blue exclusion assay showed that BA did not increase cell death in BJ fibroblasts after 72 h. In contrast, treatment of BJ cells with doxorubicin (Dox) increased cell death. Western blot analysis showed that Dox but not BA increased cl-PARP. (**B**) Western blot showed that BA had no effect on AR protein levels in BJ cells. (**C**) Western blot showed that BA does not increase total poly-Ub proteins, unlike in BA treated LNCaP cells. (**D**) DUB assay showed that BA treatment of BJ cells had no effect on DUB activity. Control lysates pre-treated with NEM for 1 h resulted in decreased DUB activity.

BA is reported to be a more selective agent against cancer compared to normal cells but the mechanisms of how this occurs have not been determined [34], [35]. Because our results in TRAMP mice showed that BA treatment did not reduce AR protein levels in non-cancerous prostate, we sought to determine the effect of BA on non-cancer cells by utilizing BJ human foreskin fibroblast cells. BJ cells treated with BA for 72 h did not increase cell death or cleaved-PARP compared to control treated cells (Fig. 7A). In contrast, treatment of BJ cells with 1 µM doxorubicin (a DNA damaging drug) resulted in substantial cell death and cleaved-PARP. Our results further showed that BA treatment did not decrease the levels of AR or increase total poly-Ub proteins in BJ cells as in LNCaP cells (Figs. 7B, C). Finally, we showed that BA had no effect on DUB activity in BJ cells, unlike what was observed in PC cells (Fig. 7D and Supplementary Fig. S8C). Similar results were obtained using human IMR-90 and MRC-5 fetal lung fibroblast cells (Supplementary Fig. S9).

**Figure 8:**
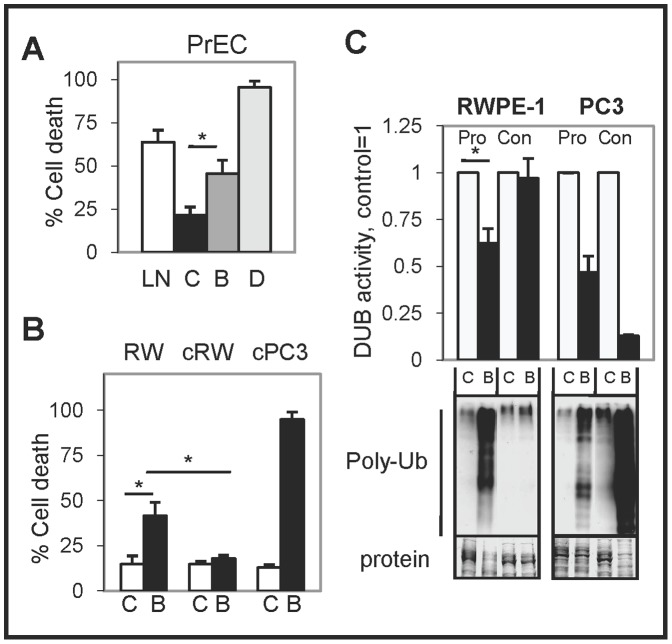
BA increases cell death and inhibits DUB activity in proliferating non-cancer prostate epithelial cells but has no effect on confluent cells. (**A**) Trypan blue exclusion assay showed that BA (B) and doxorubicin (D) increased cell death compared to vehicle (C) in PrEC cells after 72 h (*, *P*<0.003). LN was LNCaP treated with BA for 24 h. (**B**) Trypan blue exclusion assay showed that BA also increased cell death in proliferating RWPE-1 (RW) cells. However, confluent RWPE-1 (cRW) cells were more resistant to BA treatment (72 h) (*, *P*<6×10^−6^). In contrast, confluent cPC3 cells were very sensitive to BA treatment. (**C**) DUB assay and western blot showed that BA inhibited DUB activity and increased poly-Ub in proliferating (Pro) but not in confluent (Con) RWPE-1 cells (*, *P*<5×10^−4^). In PC3, BA further inhibited DUB activity and increased poly-Ub in confluent compared to proliferating cells.

Unlike in fibroblast cells, BA inhibited DUB activity and increased poly-Ub accumulation and cell death in proliferating PrEC and RWPE-1 normal prostate epithelial cells (Fig. 8). However, when RWPE-1 cells were allowed to reach confluency (high cell density) before starting BA treatment (cRWPE-1), there was no effect on DUB activity, poly-Ub accumulation, or cell death. In contrast, BA treatment of confluent PC3 cells inhibited DUB activity and increased poly-Ub accumulation greater than in proliferating PC3 cells (Fig. 8). IHC results in normal mouse tissues further demonstrated that BA treatment did not increase apoptosis or ubiquitin accumulation (Supplementary Fig. S10). Overall, these results suggested that the selectivity of BA to increase apoptotic cell death in PC cells may be due to its ability to inhibit DUBs specifically in cancer but not in non-cancer cells.

## Discussion

BA is an effective anti-cancer agent without toxicity to normal cells and tissues making it ideal for testing in PC therapy. Significantly, our results showed that BA treatment of TRAMP transgenic mice containing advanced PC inhibited tumor growth, increased apoptosis, and decreased proliferation and angiogenesis without toxicity to normal tissues. A likely mechanism for this tumor-specific effect is that BA inhibited DUB activity specifically in PC but not in normal cells, resulting in increased poly-Ub proteins and enhanced degradation of proliferation and pro-survival proteins such as cyclin D1 and AR by the UPS. Overall, these results provide evidence that BA can be an effective anti-PC agent by inhibiting multiple DUBs, which are new targets for PC therapy.

BA has potent anti-proliferation effects against the commonly used PC cell lines due to a strong induction of apoptosis (Fig. 3). Numerous other carcinoma cell lines including lung, colon, liver, pancreatic, breast, ovarian, head and neck, and renal are also inhibited by BA mainly due to induction of mitochondrial targeted apoptosis [5], [35], [36]. Our results are similar to these reports because BA increased the release of mitochondrial proteins such as cytochrome c, Smac, and AIF in PC cells (Fig. 3D). A recent report demonstrates that ectopic overexpression of Sp1 transcription factor renders pancreatic cancer cells more resistant to BA-mediated toxicity [37]. Therefore, it is likely that that the degradation of specific proliferation and pro-survival proteins individually or in combination are required for BA-mediated apoptosis, although the precise mechanisms are not clear. Alternatively, the BA-mediated pro-apoptotic death signal may originate from the accumulation of poly-Ub proteins resulting from the inhibition of multiple DUBs [38].

In LNCaP cells, the dependence of BA on the UPS pathway for increased protein degradation was supported by results demonstrating that 1) a cyclin D1 mutant that cannot be degraded by the UPS was resistant to BA-mediated degradation; and 2) the UPS inhibitor MG132 blocked the BA-mediated decrease in AR, AKT, and Mcl-1. Since BA is reported to increase the degradation of multiple members of the Sp transcription factor family in PC cells, transcription of proliferation and pro-survival genes dependent on Sp may also be further reduced [8]. Therefore, the BA-mediated degradation of Sp transcription factors likely amplifies the UPS-mediated decrease in the expression of proliferation and pro-survival genes. Similar to our *in vitro* results in PC cell lines, BA treatment decreased AR and cyclin D1 protein levels and increased total Ub proteins in TRAMP tumors. A chemotherapy agent such as BA that can specifically degrade AR and cyclin D1 is especially important in PC therapy due to the importance of these proteins in tumor progression. AR is the most important factor for the emergence of CRPC and cyclin D1 has a role in PC progression, regulation of AR activity, and may be a significant prognostic marker for aggressive metastatic PC [39]–[42].

The mechanism why BA increased the degradation of cell cycle and pro-survival proteins was likely by the inhibition of multiple DUBs, which resulted in increased levels of total poly-Ub proteins that are recognized by the UPS and degraded. Our preliminary data suggests that BA specifically inhibits USP7, 9x, and 10 in PC3 cells (data not shown). USP7, also known as herpesvirus-associated ubiquitin-specific protease or HAUSP, is overexpressed in PC, deubiquitinates PTEN protein to block its function, and regulates p53 levels [43], [44]. USP9x regulates the levels of the anti-apoptotic protein Mcl-1 and has a role in TGFβ signaling by controlling Smad4 mono-Ub [45], [46]. At present, the DUB that regulates AR protein levels is not known. USP10 is an AR cofactor important for activation of AR regulated genes [47], [48]. However, it is not clear if inhibition or shRNA knockdown of USP10 lowers AR protein levels. Once the DUB(s) that regulate AR protein levels are identified, this should provide new high impact drug target(s) for the identification of other small molecule inhibitors in addition to BA that could have therapeutic efficacy in PC.

An alternative strategy to target the UPS without the toxic side effects to normal cells may be by inhibiting upstream DUB activity. One possible mechanism for BA's lack of toxicity to non-cancer cells is that BA had no effect on DUB activity in normal cells such as BJ, IMR-90, MRC-5 fibroblasts and confluent RWPE-1 prostate epithelial cells compared to PC cells (Figs. 6–8; supplemental Fig. S9). The reason for this difference is not clear but may reflect that DUBs are often overexpressed in cancer cells [9]–[11]. Interestingly, the DUB labeling assay revealed only two DUBs present in PC but not in BJ cells (bands 2 and 3, Supplementary Fig. S8D); other DUB labeled bands that appeared more highly expressed in PC compared to BJ cells include bands 1, 4, and 5. We are currently investigating the identities of the DUB family members inhibited by BA and play a role in sensitizing PC cells to BA treatment without harming normal cells. Alternatively, the reason BA inhibits DUB activity in PC but not in non-cancer cells may be due to an indirect mechanism.

Inhibition of the UPS pathway for anti-cancer chemotherapy has led to the successful development of bortezomib [3]. This report addressed the utilization of a small molecule such as BA to increase the degradation of proliferation and pro-survival proteins via the UPS by inhibiting upstream DUB activity in PC cells. Further investigation will be required to determine whether BA directly or indirectly inhibits DUB activity in PC cells. In contrast, BA had no effect on DUB activity in normal fibroblast and confluent prostate epithelial cells, perhaps explaining why BA was non-toxic to normal cells and tissues. Since DUBs are overexpressed in PC cells, they provide a new target that will result in an increase in therapeutic efficacy by reducing pro-survival proteins such as AR. Our data suggests that BA-mediated inhibition of DUBs and induction of apoptotic cell death specifically in PC but not in non-cancer cells will provide an effective non-toxic and clinically selective agent for chemotherapy.

## Supporting Information

Figure S1 **BA treatment of TRAMP mice with prostate tumors increases apoptosis and decreases angiogenesis and proliferation.** Representative immunostaining for cleaved (cl)-caspase-3 and TUNEL (apoptosis), CD31 and CD34 (angiogenesis), and Ki67 and PCNA (proliferation) in prostate tumors from TRAMP mice treated with vehicle control or BA10 (×200).
(TIF)

Figure S2 **BA increases the release of mitochondrial proteins in LNCaP and DU145 cells.** Mitochondrial protein release assay and western blot analysis showed increased levels of cytochrome c, Smac, and AIF in LNCaP and DU145 cells treated with BA compared to control (0 hrs) cells. Cox IV protein was negative or weak indicating no or minimal mitochondrial contamination whereas actin was the positive control. Coomassie blue stain of total protein was loading control. +C was lysate prepared using the standard method for total proteins.
(TIF)

Figure S3 **(A) BA increased G1/S cell cycle block in PC cells. Flow cytometric analysis of LNCaP,** DU145, and PC3 treated with BA or control for 24 h resulted in increased cells in G1 and decreased cells in S phase. Numbers in parenthesis are the percentage of cells in each cell cycle phase from three independent experiments done in duplicate. There was no change in G2/M and increased sub (s)-G1. (**B**) BA increased cells in the sub-G1 cell cycle phase at later time points. Flow cytometric analysis of LNCaP, DU145, and PC3 treated with BA for 48 (LN) or 72 h (DU/PC) showed increased cells in sub-G1, indicating DNA breakage. In DU145 and PC3 but not in LNCaP cells, there was significantly increased cells in G2/M. Numbers in parenthesis were the percentage of cells in each cell cycle phase from three independent experiments done in duplicate.
(TIF)

Figure S4 **BA increases the degradation of multiple cell cycle and pro-survival proteins in DU145 cells.** Western blot analysis showed that BA treatment resulted in lower protein levels of cyclins, Cdks, E2F1, Rb, AR (transfected), AKT, and survivin and higher levels of p27 and cl-PARP in DU145 cells, similar to results in LNCaP and PC3 cells. BA treatment also decreased the levels of mutant p53 protein. Unlike in LNCaP and PC3 cells, BA treatment of DU145 cells did not decrease Mcl-1 protein.
(TIF)

Figure S5 UPS assay showed significantly increased proteasome activity in DU145 and PC3 cells treated with BA for 24, 48, and 72 h (*, ***P*** <0.03; **, ***P*** <0.003; ***, ***P*** **<3×10^−5^).** Addition of MG132 (MG) to BA resulted in decreased proteasome activity.
(TIF)

Figure S6 **(A) BA treatment of TRAMP mice with prostate tumors increased immunostaining for ubiquitin compared to vehicle control (×200).** (**B**) DUB assay showed that BA10 (n = 5) significantly decreased DUB activity in TRAMP prostate tumors relative to vehicle control (n = 4) (*, *P*<6×10^−5^). Control lysates pre-treated with 4 mM NEM for 1 h resulted in decreased DUB activity.
(TIF)

Figure S7 **DUB assay showed that BA treatment of DU145 and PC3 cells significantly decreased DUB activity at 48 and 72 h relative to control treated cells ( = 1).** Unlike in LNCaP, treatment of DU145 and PC3 with 1 nM Doc also reduced DUB activity, although not as great as in BA treated cells (*, *P*<0.05; **, *P*<7×10^−3^). Control lysates pre-treated with 4 mM NEM for 1 h resulted in decreased DUB activity.
(TIF)

Figure S8 **DUB activity labeling with HA-UbVS showed that BA but not Doc inhibited multiple DUBs in DU145 (A) and PC3 (B) cells.** Protein bands 1–5 showed the strongest decrease in activity with BA treatment. In contrast, BA does not inhibit DUB activity in BJ (**C**) cells. Control lysates without addition of HA-UbVS or pre-incubated with NEM for 1 h were the controls. (**D**) Control LNCaP (LN), LN-AI (AI, androgen-independent variant of LNCaP), DU (DU145), PC (PC3) cells labeled with HA-UbVS are compared with BJ cells. Molecular weight markers (kDa) are shown to the left. Coomassie blue stain of total protein were loading controls.
(TIF)

Figure S9 **BA had no effect on non-cancer IRM-90 and MRC-5 fetal lung fibroblasts.** (**A**) Trypan blue exclusion assay showed that BA did not increase cell death in IRM-90 and MRC-5 after 72 h. In contrast, doxorubicin (Dox) increased cell death (n = 7–11, three experiments). (**B**) Western blot analysis showed that Dox but not BA increased cl-PARP. +C is LNCaP BA 24 h. (**C**) In contrast to LNCaP (+C), BA did not increase poly-Ub accumulation in MRC-5 and IRM-90. (**D**) DUB assay showed that BA treatment of IRM-90 cells had no effect on DUB activity. Control lysates pre-treated with NEM for 1 h resulted in decreased DUB activity (n = 6, two experiments).
(TIF)

Figure S10 **BA did not increase apoptosis or ubiquitin in normal mouse tissue.** IHC (×200) results showed little difference between BA10 and vehicle control TRAMP spleen and thymus for cleaved caspase-3 (apoptosis) and ubiquitin.
(TIF)
