# Supplementary figures and images for: Betulinic Acid Selectively Increases Protein Degradation and Enhances Prostate Cancer-Specific Apoptosis: Possible Role for Inhibition of Deubiquitinase Activity
Source: PLoS One. 2013 Feb 12;8(2):e56234. doi: 10.1371/journal.pone.0056234 (PMC3570422; doi:10.1371/journal.pone.0056234)

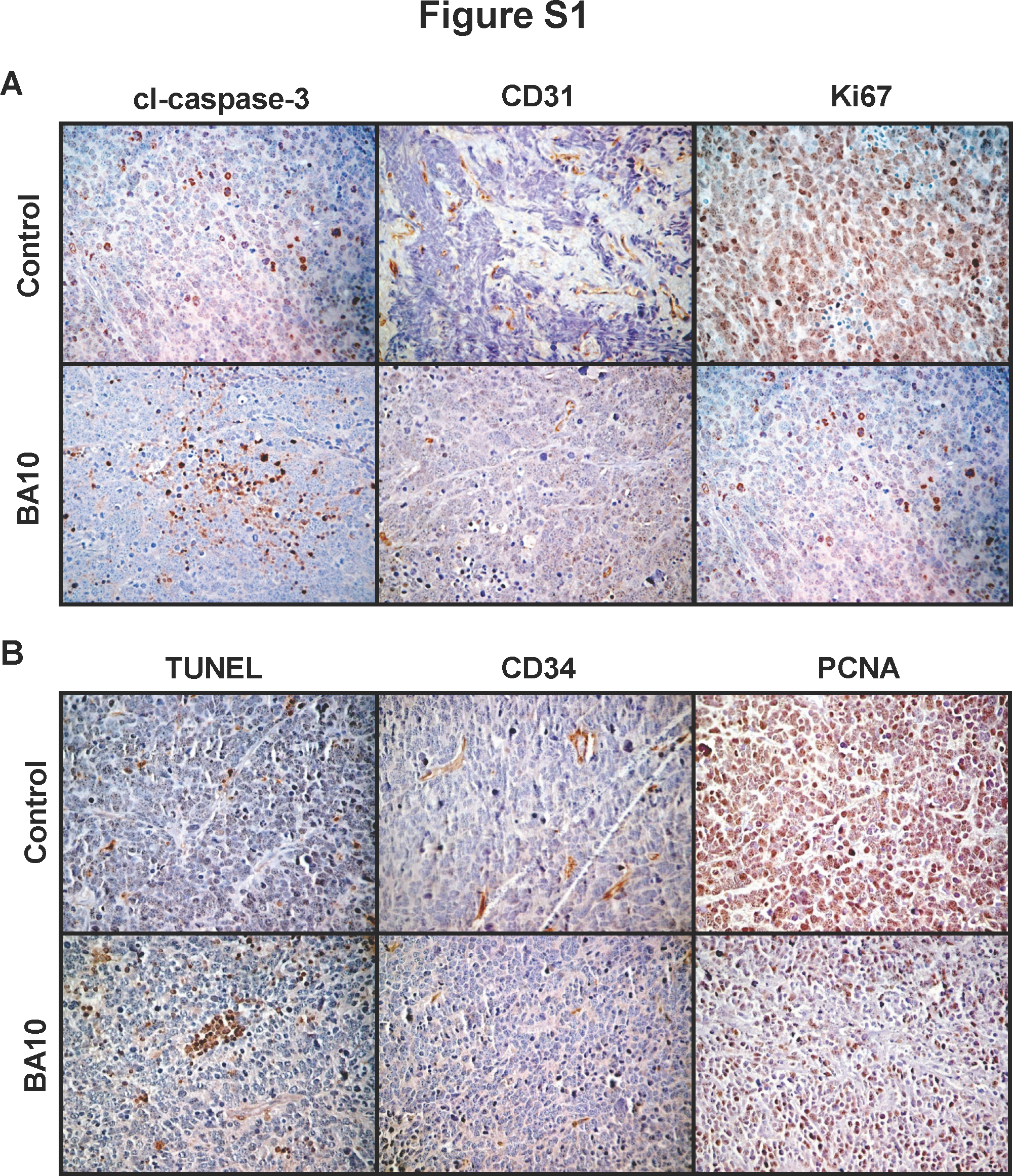

Supplement: Figure S1 — BA treatment of TRAMP mice with prostate tumors increases apoptosis and decreases angiogenesis and proliferation. Representative immunostaining for cleaved (cl)-caspase-3 and TUNEL (apoptosis), CD31 and CD34 (angiogenesis), and Ki67 and PCNA (proliferation) in prostate tumors from TRAMP mice treated with vehicle control or BA10 (×200). (TIF) [file pone.0056234.s001.tif]

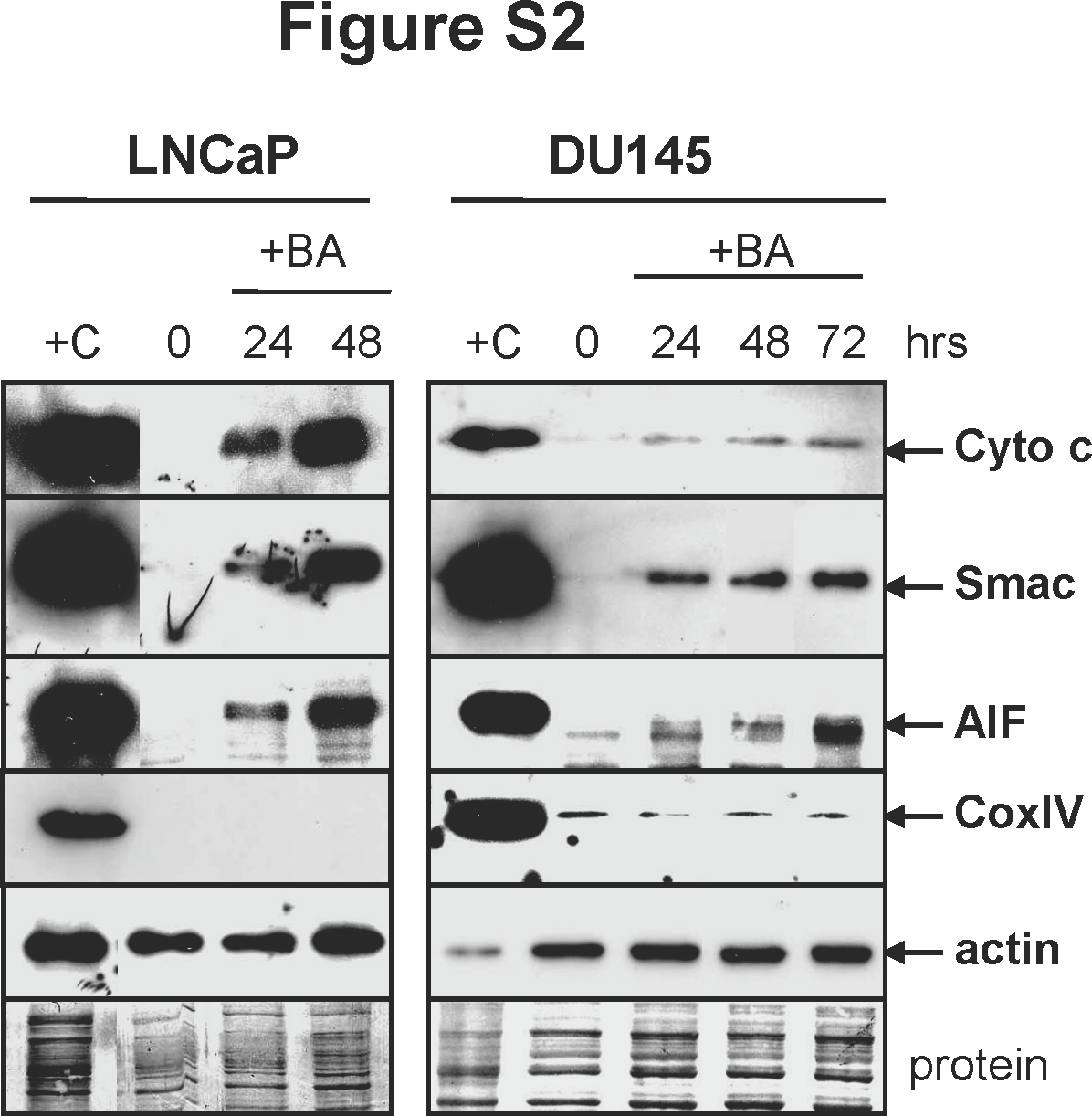

Supplement: Figure S2 — BA increases the release of mitochondrial proteins in LNCaP and DU145 cells. Mitochondrial protein release assay and western blot analysis showed increased levels of cytochrome c, Smac, and AIF in LNCaP and DU145 cells treated with BA compared to control (0 hrs) cells. Cox IV protein was negative or weak indicating no or minimal mitochondrial contamination whereas actin was the positive control. Coomassie blue stain of total protein was loading control. +C was lysate prepared using the standard method for total proteins. (TIF) [file pone.0056234.s002.tif]

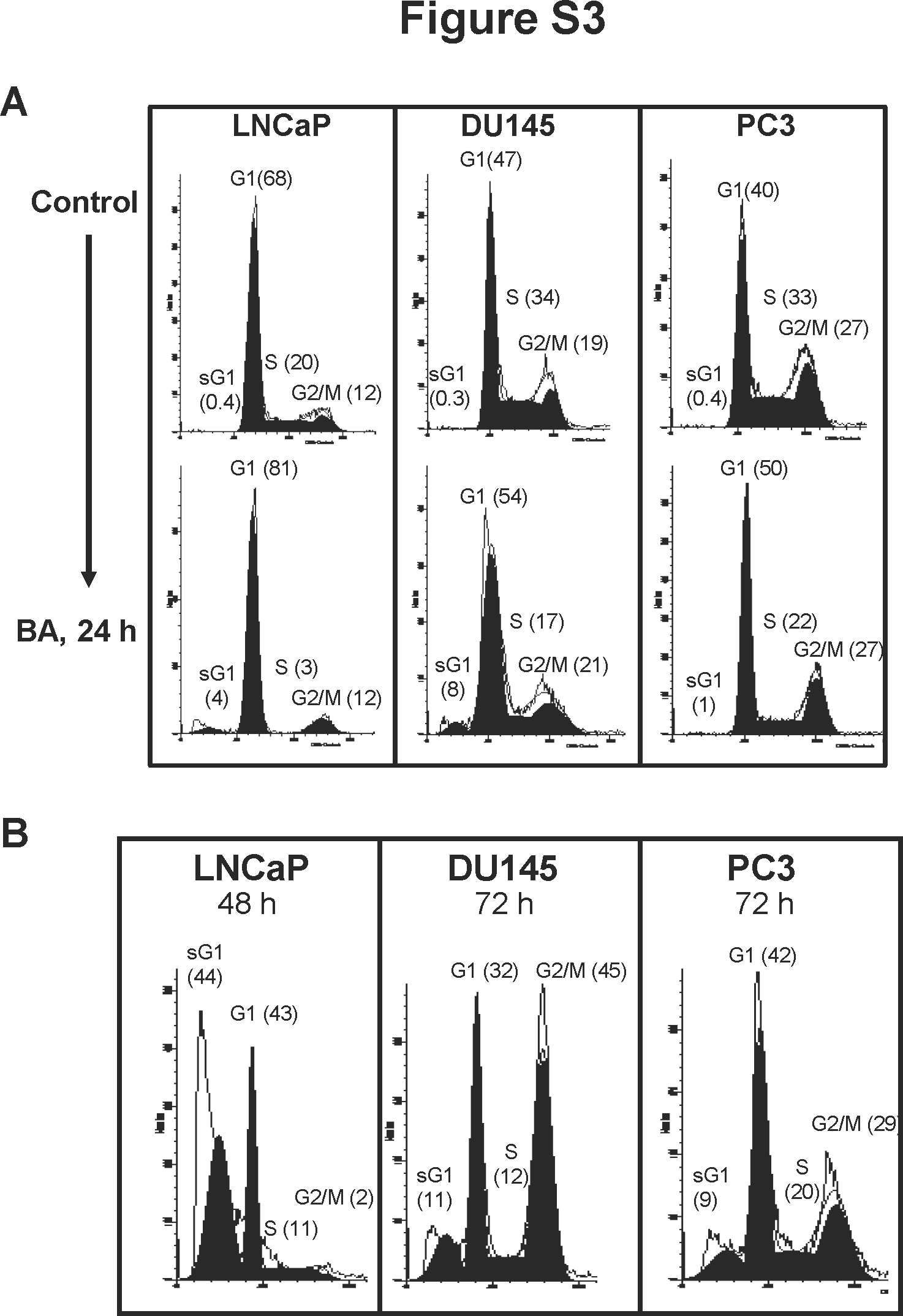

Supplement: Figure S3 — (A) BA increased G1/S cell cycle block in PC cells. Flow cytometric analysis of LNCaP, DU145, and PC3 treated with BA or control for 24 h resulted in increased cells in G1 and decreased cells in S phase. Numbers in parenthesis are the percentage of cells in each cell cycle phase from three independent experiments done in duplicate. There was no change in G2/M and increased sub (s)-G1. (B) BA increased cells in the sub-G1 cell cycle phase at later time points. Flow cytometric analysis of LNCaP, DU145, and PC3 treated with BA for 48 (LN) or 72 h (DU/PC) showed increased cells in sub-G1, indicating DNA breakage. In DU145 and PC3 but not in LNCaP cells, there was significantly increased cells in G2/M. Numbers in parenthesis were the percentage of cells in each cell cycle phase from three independent experiments done in duplicate. (TIF) [file pone.0056234.s003.tif]

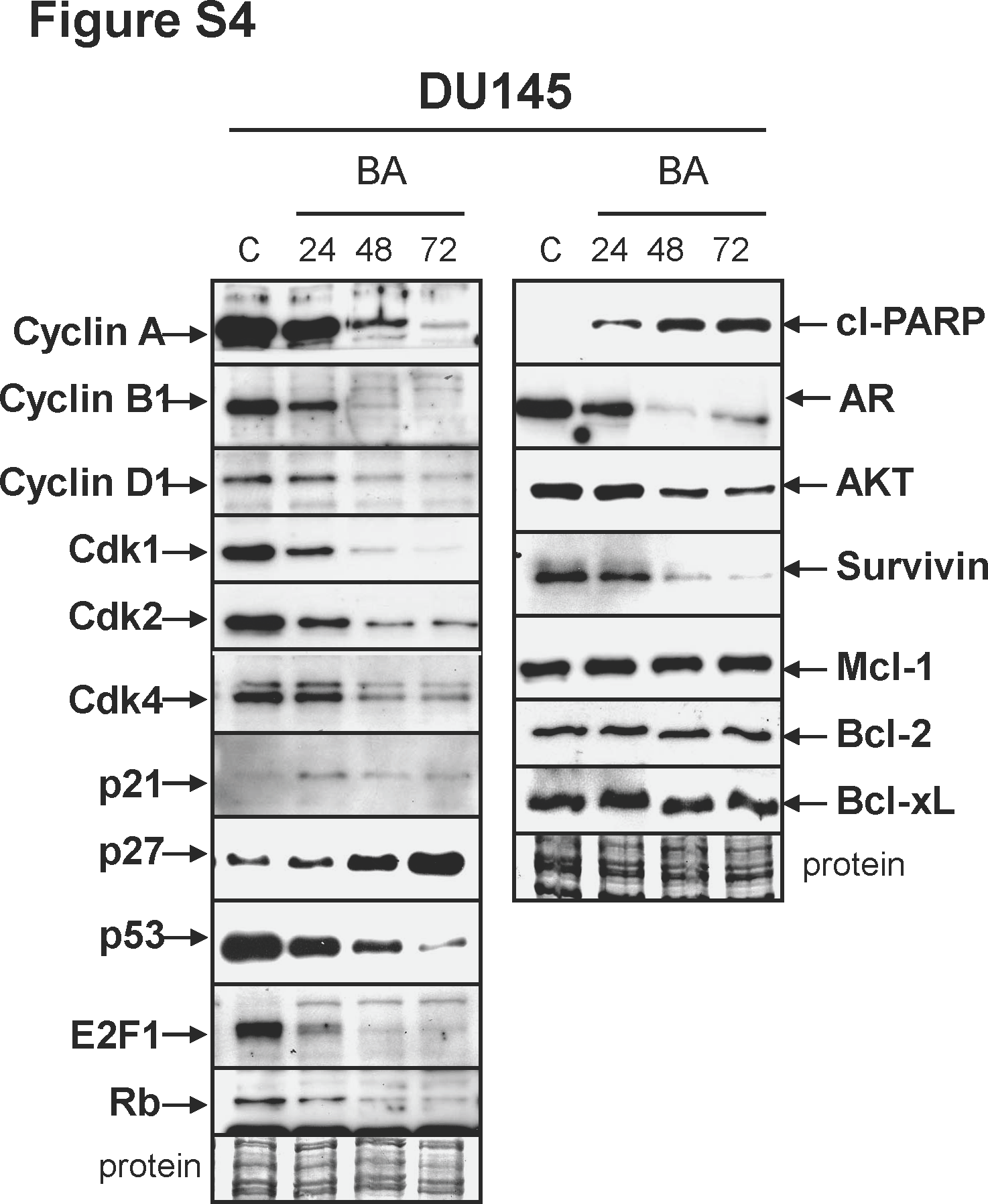

Supplement: Figure S4 — BA increases the degradation of multiple cell cycle and pro-survival proteins in DU145 cells. Western blot analysis showed that BA treatment resulted in lower protein levels of cyclins, Cdks, E2F1, Rb, AR (transfected), AKT, and survivin and higher levels of p27 and cl-PARP in DU145 cells, similar to results in LNCaP and PC3 cells. BA treatment also decreased the levels of mutant p53 protein. Unlike in LNCaP and PC3 cells, BA treatment of DU145 cells did not decrease Mcl-1 protein. (TIF) [file pone.0056234.s004.tif]

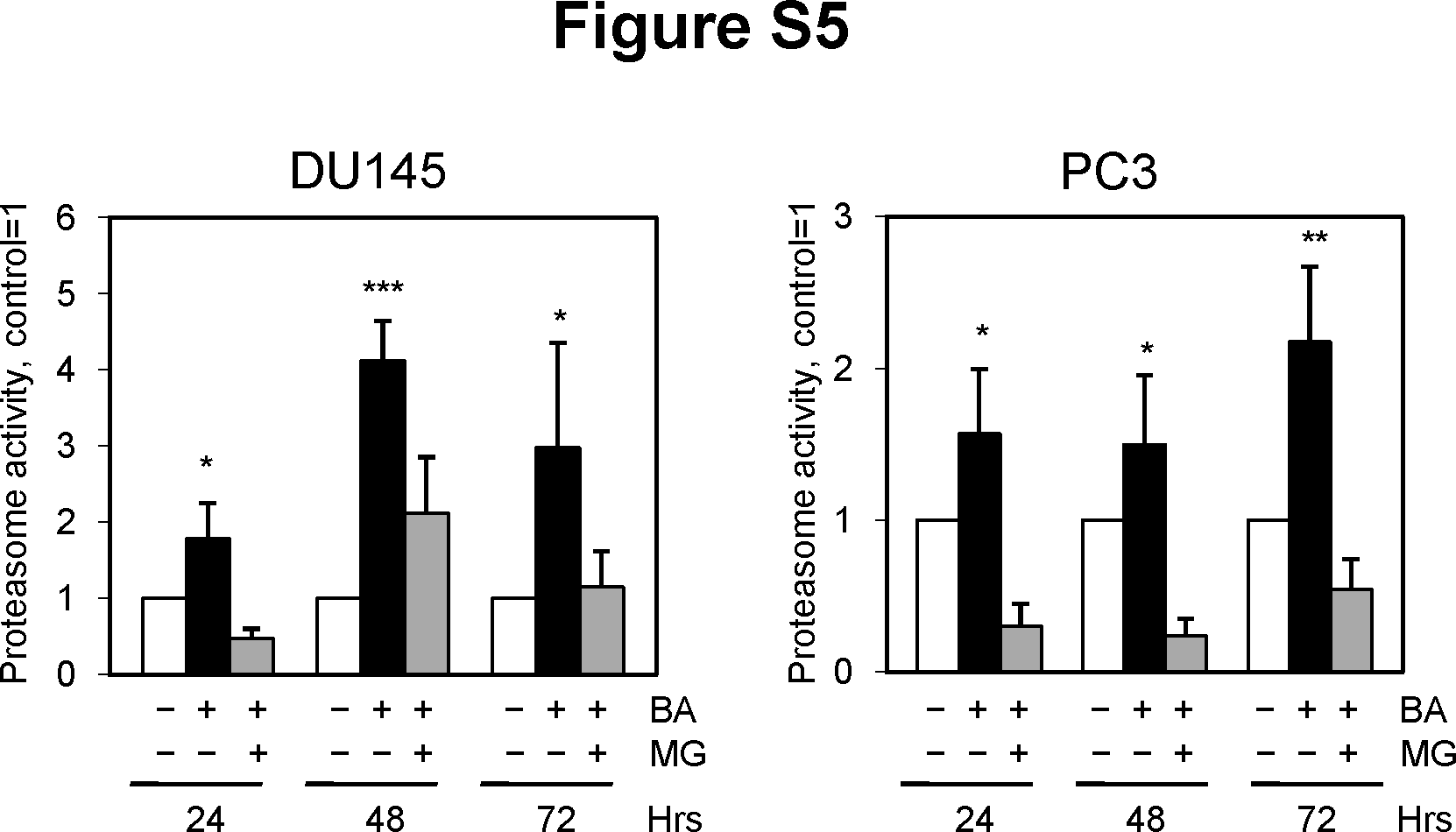

Supplement: Figure S5 — UPS assay showed significantly increased proteasome activity in DU145 and PC3 cells treated with BA for 24, 48, and 72 h (*, P <0.03; **, P <0.003; ***, P <3×10^−5^). Addition of MG132 (MG) to BA resulted in decreased proteasome activity. (TIF) [file pone.0056234.s005.tif]

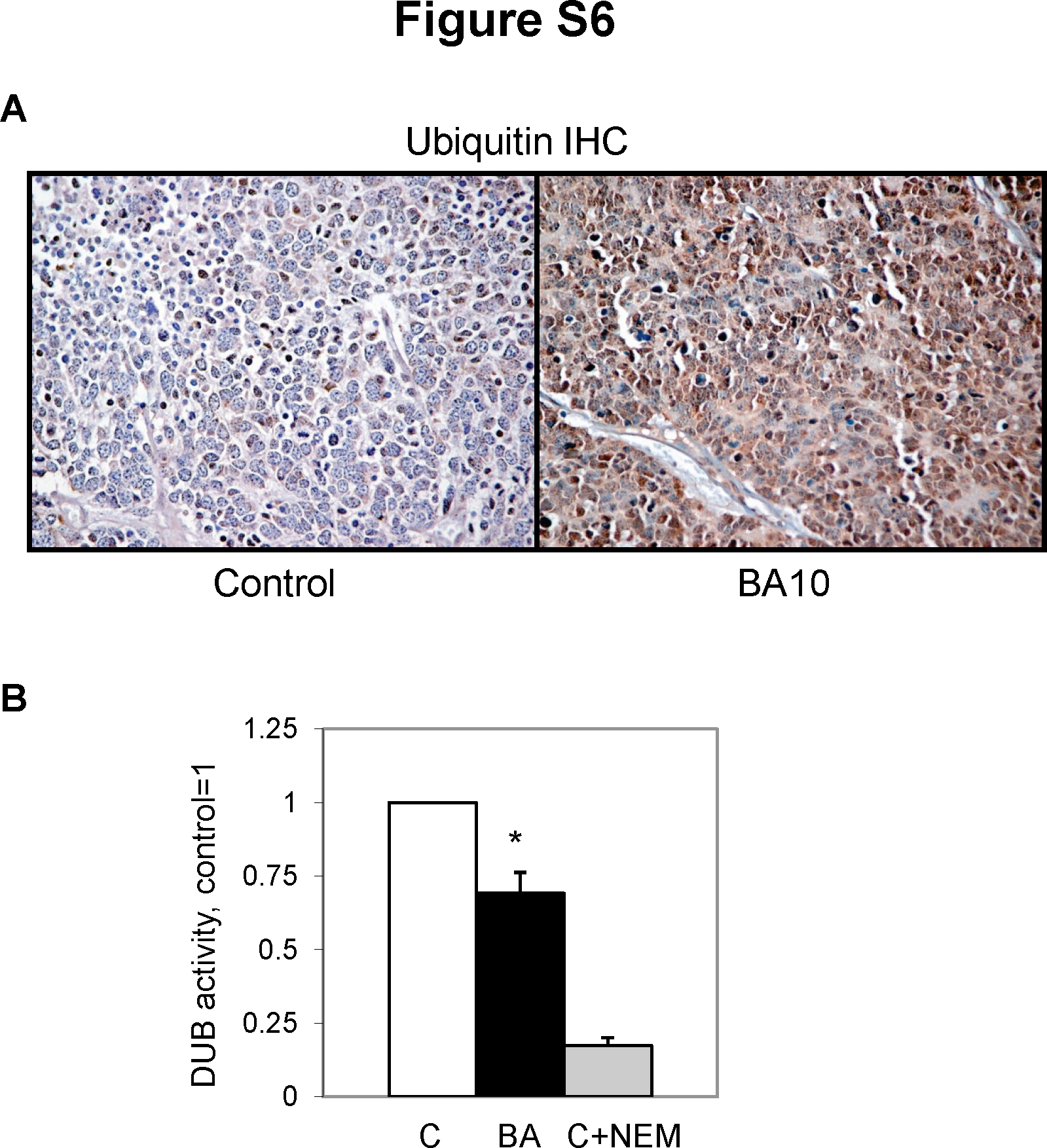

Supplement: Figure S6 — (A) BA treatment of TRAMP mice with prostate tumors increased immunostaining for ubiquitin compared to vehicle control (×200). (B) DUB assay showed that BA10 (n = 5) significantly decreased DUB activity in TRAMP prostate tumors relative to vehicle control (n = 4) (*, P<6×10^−5^). Control lysates pre-treated with 4 mM NEM for 1 h resulted in decreased DUB activity. (TIF) [file pone.0056234.s006.tif]

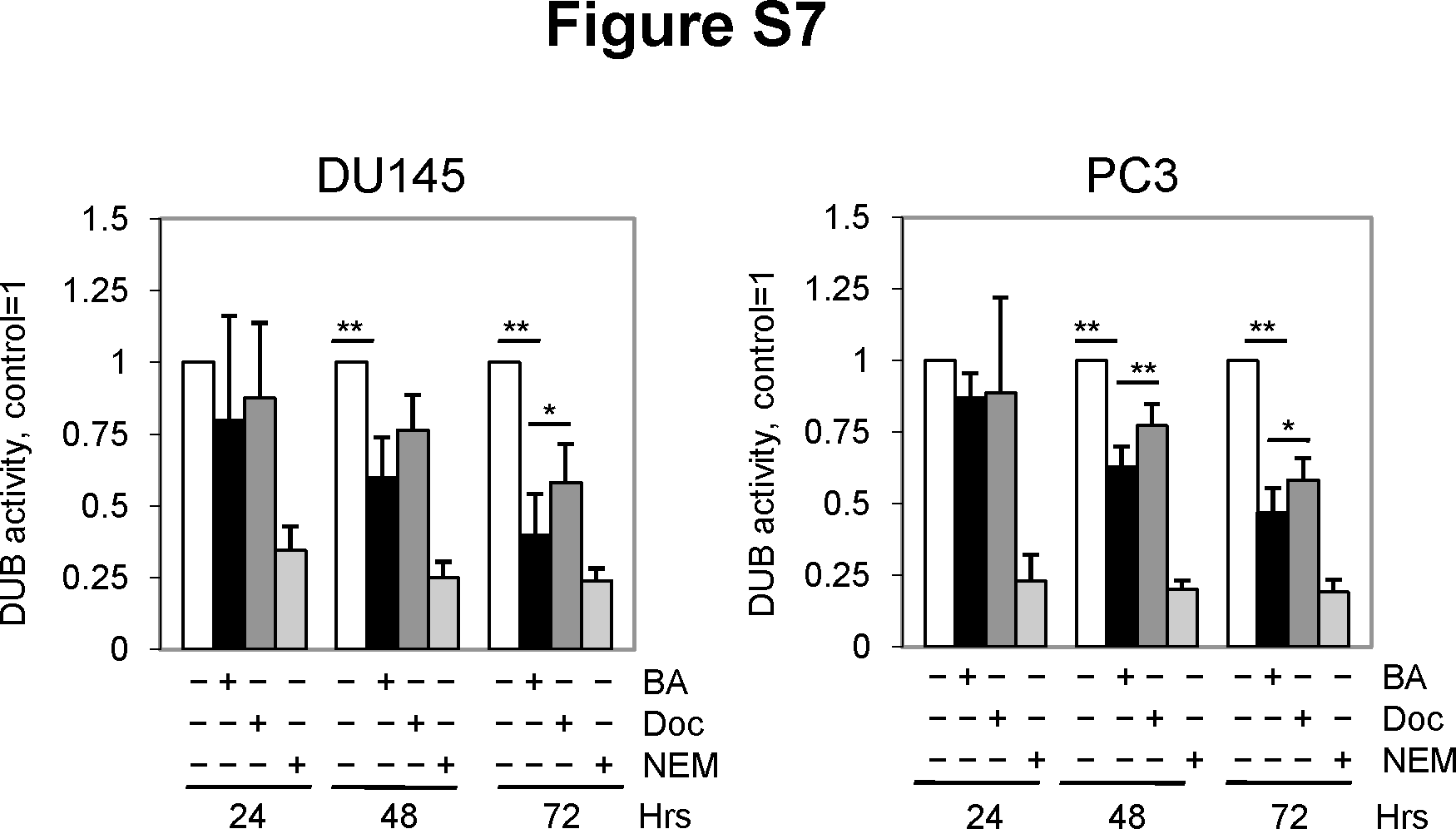

Supplement: Figure S7 — DUB assay showed that BA treatment of DU145 and PC3 cells significantly decreased DUB activity at 48 and 72 h relative to control treated cells ( = 1). Unlike in LNCaP, treatment of DU145 and PC3 with 1 nM Doc also reduced DUB activity, although not as great as in BA treated cells (*, P<0.05; **, P<7×10^−3^). Control lysates pre-treated with 4 mM NEM for 1 h resulted in decreased DUB activity. (TIF) [file pone.0056234.s007.tif]

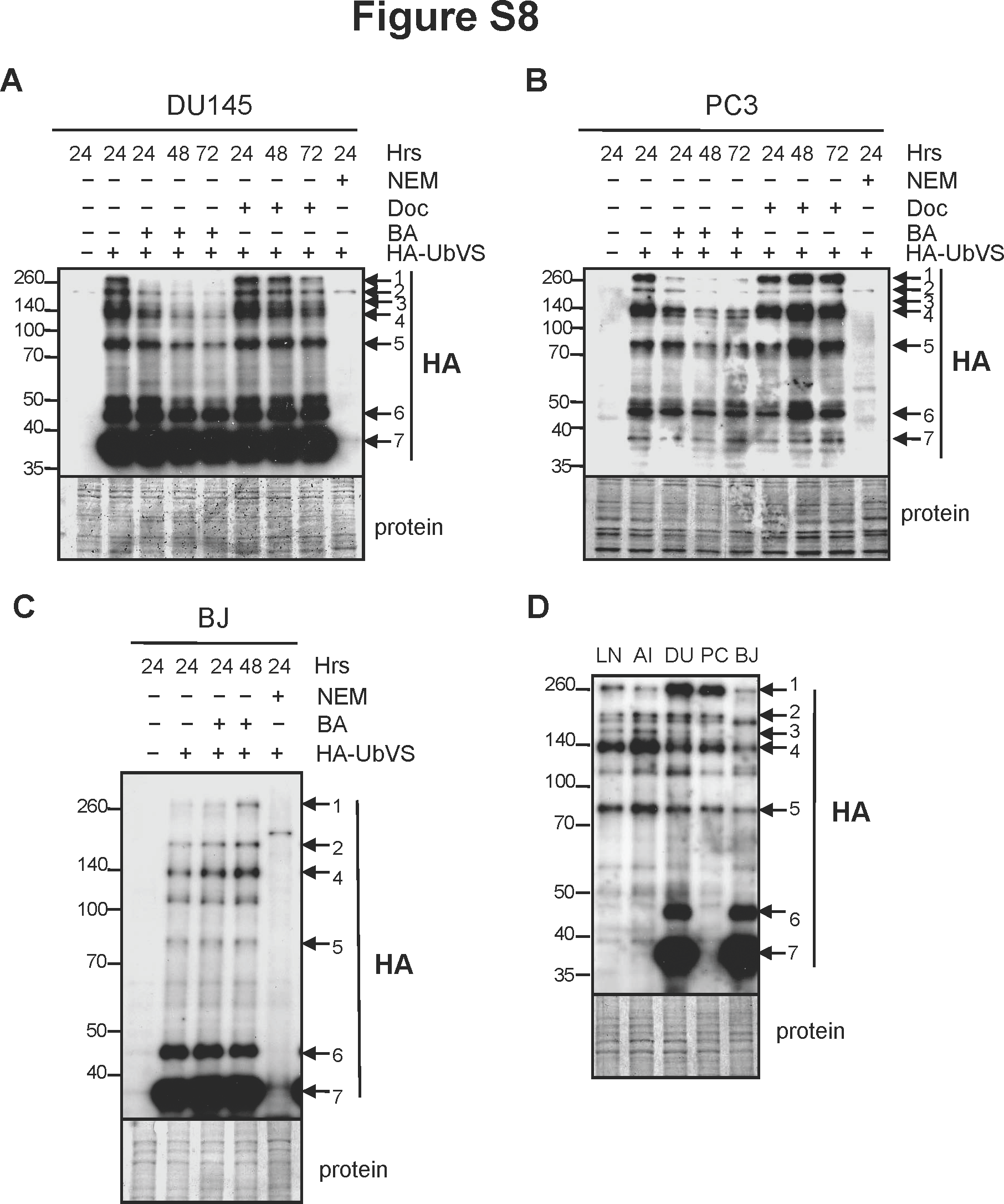

Supplement: Figure S8 — DUB activity labeling with HA-UbVS showed that BA but not Doc inhibited multiple DUBs in DU145 (A) and PC3 (B) cells. Protein bands 1–5 showed the strongest decrease in activity with BA treatment. In contrast, BA does not inhibit DUB activity in BJ (C) cells. Control lysates without addition of HA-UbVS or pre-incubated with NEM for 1 h were the controls. (D) Control LNCaP (LN), LN-AI (AI, androgen-independent variant of LNCaP), DU (DU145), PC (PC3) cells labeled with HA-UbVS are compared with BJ cells. Molecular weight markers (kDa) are shown to the left. Coomassie blue stain of total protein were loading controls. (TIF) [file pone.0056234.s008.tif]

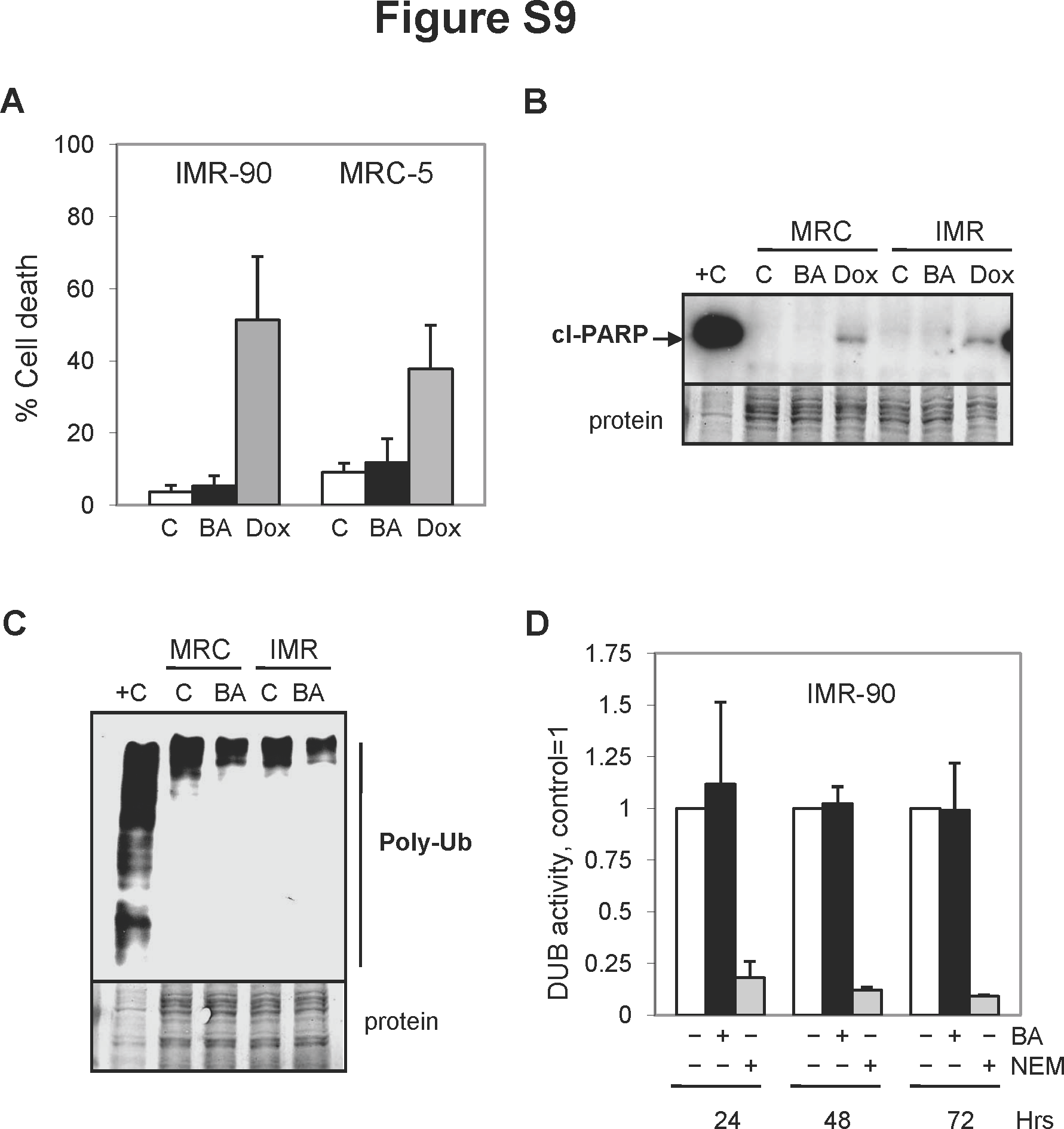

Supplement: Figure S9 — BA had no effect on non-cancer IRM-90 and MRC-5 fetal lung fibroblasts. (A) Trypan blue exclusion assay showed that BA did not increase cell death in IRM-90 and MRC-5 after 72 h. In contrast, doxorubicin (Dox) increased cell death (n = 7–11, three experiments). (B) Western blot analysis showed that Dox but not BA increased cl-PARP. +C is LNCaP BA 24 h. (C) In contrast to LNCaP (+C), BA did not increase poly-Ub accumulation in MRC-5 and IRM-90. (D) DUB assay showed that BA treatment of IRM-90 cells had no effect on DUB activity. Control lysates pre-treated with NEM for 1 h resulted in decreased DUB activity (n = 6, two experiments). (TIF) [file pone.0056234.s009.tif]

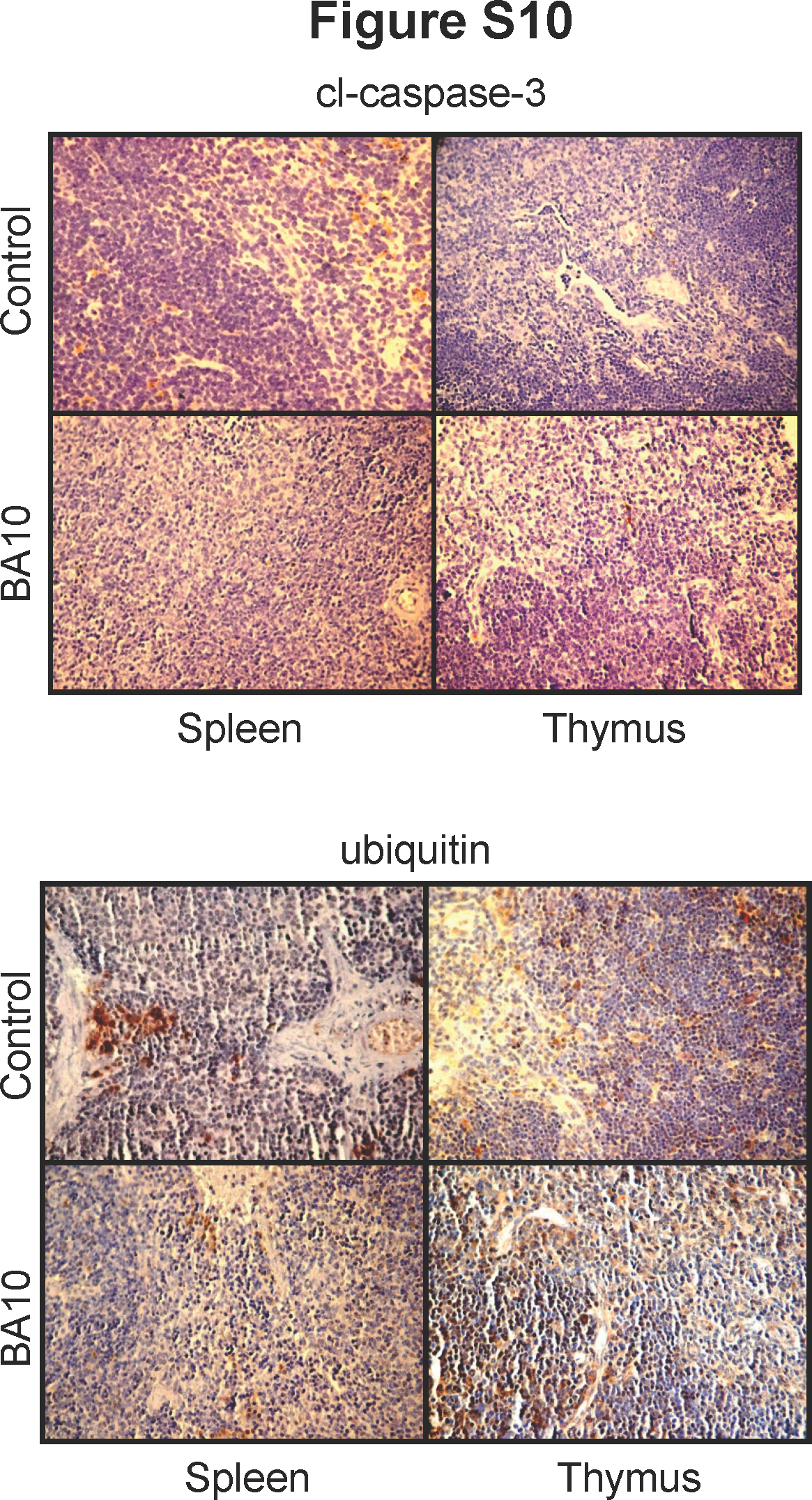

Supplement: Figure S10 — BA did not increase apoptosis or ubiquitin in normal mouse tissue. IHC (×200) results showed little difference between BA10 and vehicle control TRAMP spleen and thymus for cleaved caspase-3 (apoptosis) and ubiquitin. (TIF) [file pone.0056234.s010.tif]
